# A Retrospective Study for the Evaluation of Serum Anti-Müllerian Hormone and Its Correlation With Insulin Resistance and Lipid Profile in Polycystic Ovary Syndrome in Adolescents

**DOI:** 10.7759/cureus.94344

**Published:** 2025-10-11

**Authors:** Tahsina Begum, J. Ashwini Kumari, Naaz Fatima, Noorjahan Mohammed, Radhika Gollapudi

**Affiliations:** 1 Biochemistry, MNR Medical College and Hospital, Hyderabad, IND; 2 Biochemistry, Gandhi Medical College, Hyderabad, IND; 3 Obstetrics and Gynaecology, Mahavir Institute of Medical Sciences, Hyderabad, IND; 4 Biochemistry, Nizam's Institute of Medical Sciences, Hyderabad, IND; 5 Obstetrics and Gynecology, Nizam's Institute of Medical Sciences, Hyderabad, IND

**Keywords:** anti-müllerian hormone, homa-ir, insulin resistance, luteinizing hormone, polycystic ovary syndrome

## Abstract

Background and rationale

Polycystic ovary syndrome (PCOS) is a prevalent endocrine disorder in adolescents, marked by hormonal imbalances, insulin resistance, and metabolic dysfunction. Early identification of reliable biochemical markers is crucial for timely diagnosis and management to prevent long-term complications like type 2 diabetes and cardiovascular disease. This study investigates serum anti-Müllerian hormone (AMH) and its correlation with insulin resistance and lipid profiles to identify predictive markers for PCOS risk in adolescents.

Methods

A retrospective study analyzed 91 women (49 PCOS cases per Rotterdam criteria, 42 age-matched controls; mean age 25.5 ± 5.3 years). Biochemical parameters, including AMH, insulin, fasting blood sugar (FBS), luteinizing hormone (LH), interleukin-6 (IL-6), lipid profiles (total cholesterol (TC), high-density lipoprotein (HDL), low-density lipoprotein (LDL), triglycerides (TG)), and electrolytes (sodium, chloride), were measured using standardized protocols on the Beckman Coulter AU series autoanalyzer. Insulin resistance was assessed via Homeostatic Model Assessment for Insulin Resistance (HOMA-IR) and Quantitative Insulin Sensitivity Check Index (QUICKI). Statistical analyses included t-tests, logistic regression, exploratory factor analysis (EFA), and heatmap visualization of PCOS phenotypes (Groups A-D, based on clinical/biochemical severity).

Results

The PCOS group showed significantly higher insulin (mean difference: 5.2 µIU/mL, p = 0.003), HOMA-IR (p = 0.004), LH (p = 0.002), AMH (p < 0.001), and chloride (p < 0.001), with lower Na:Cl ratio (p < 0.001) and QUICKI (p = 0.005) compared to controls. Logistic regression identified AMH (odds ratio (OR): 1.45, p < 0.001), FBS (OR: 1.12, p = 0.02), and LH (OR: 1.33, p = 0.01) as key predictors. Model 5 (AMH, FBS, IL-6, LH) achieved a McFadden R² of 0.85 and area under the curve (AUC) of 1.00. EFA revealed five factors (36.4% variance): insulin resistance, liver/lipid function, lipid metabolism, liver/kidney function, and electrolyte/protein balance, all correlated with PCOS (p < 0.05). Heatmap analysis showed severe insulin resistance in Group D and elevated FBS/chloride in Group C.

Conclusion

AMH, LH, and FBS are robust markers for diagnosing and managing PCOS in adolescents. These, alongside insulin resistance and lipid alterations, support early risk stratification and personalized interventions across PCOS phenotypes, improving long-term health outcomes.

## Introduction

Polycystic ovary syndrome (PCOS) affects 6-20% of women of reproductive age, significantly impacting metabolic, reproductive, and psychological health [1]. In adolescents, PCOS manifests as hyperandrogenism, ovulatory dysfunction, and polycystic ovarian morphology, often accompanied by insulin resistance, dyslipidemia, and elevated inflammatory markers [2]. Early identification of biochemical markers is crucial for timely diagnosis to prevent complications such as type 2 diabetes, cardiovascular disease, and infertility [3,4].

Key markers, including luteinizing hormone (LH), anti-Müllerian hormone (AMH), insulin, and Homeostatic Model Assessment for Insulin Resistance (HOMA-IR), reflect disruptions in the hypothalamic-pituitary-ovarian axis and metabolic homeostasis [5]. AMH, a marker of ovarian follicular reserve, is elevated in PCOS due to increased number of antral follicles, making it a promising diagnostic tool [6]. Inflammatory markers like interleukin-6 (IL-6), metabolic indicators such as fasting blood sugar (FBS), and lipid profiles contribute to PCOS pathophysiology, although their utility in adolescents is less established [7,8]. Emerging evidence suggests liver function tests (e.g., serum glutamic oxaloacetic transaminase (SGOT)) and electrolyte imbalances (e.g., chloride, Na:Cl ratio) may reflect systemic metabolic stress, which can enhance diagnostic panels [9,10].

This study aimed to: (1) compare biochemical markers between women with and without PCOS; (2) develop predictive models for PCOS detection using logistic regression; (3) explore metabolic changes across PCOS phenotypes (Groups A-D) using median-based heatmap analysis; and (4) explore the underlying structure of these markers through exploratory factor analysis (EFA). We hypothesized that women with PCOS would exhibit elevated insulin, HOMA-IR, LH, AMH, and inflammatory markers, serving as robust predictors for clinical diagnosis, with phenotype-specific metabolic variations, particularly in insulin resistance and electrolyte balance.

## Materials and methods

Study design

A total of 91 women (49 PCOS cases, 42 controls; mean age 25.45 ± 5.55 years for cases, 26.91 ± 5.01 years for controls) were recruited from a tertiary care hospital in an urban setting during the period of October 2024 to March 2025. Power analysis indicated > 80% power for key variables (AMH, LH, FBS, insulin, HOMA-IR, chloride), supporting the reliability of observed associations. PCOS diagnosis was based on the Rotterdam criteria, requiring at least two of the following: oligo-/anovulation, clinical/biochemical hyperandrogenism, or polycystic ovaries on ultrasound. PCOS cases were retrospectively classified into four phenotypes based on Rotterdam criteria and clinical data: Phenotype A: Hyperandrogenism + Ovulatory dysfunction + Polycystic ovaries (n = 14, 28.6%); Phenotype B: Hyperandrogenism + Ovulatory dysfunction (n=18, 36.7%); Phenotype C: Hyperandrogenism + Polycystic ovaries (n = 5, 10.2%); Phenotype D: Ovulatory dysfunction + Polycystic ovaries (n = 12, 24.5%). This stratification aligns with adolescent PCOS prevalence patterns reported in South Asia. Controls were confirmed to be free of PCOS based on both transabdominal ultrasound and hormonal profiling. Informed consent was obtained from participants and their legal guardians, with approval from the institutional ethics review board.

All assays were performed using standardized, manufacturer-recommended protocols on the Beckman Coulter AU series clinical chemistry autoanalyzer (e.g., AU480 or AU5800 model), which employs photometric and enzymatic methodologies for high-throughput, automated processing. This system supports a full menu of over 120 assays, including enzymatic colorimetric methods for lipids (e.g., cholesterol oxidase for total cholesterol (TC), precipitation for HDL, and glycerol blanking for triglycerides (TG)), kinetic ultraviolet assays for liver enzymes (e.g., International Federation of Clinical Chemistry and Laboratory Medicine (IFCC) method for aspartate aminotransferase (AST)/alanine aminotransferase (ALT)), Jaffe kinetic method for creatinine, urease-Berthelot method for urea, ion-selective electrode (ISE) technology for electrolytes (sodium, potassium, chloride), and diazo method for bilirubin fractions. The analyzer features onboard sample dilution, auto-verification of results, and integration with laboratory information systems for seamless workflow.

For glycemic and lipid parameters (FBS, TC, high-density lipoprotein (HDL), low-density lipoprotein (LDL), very low-density lipoprotein (VLDL), TG), enzymatic assays were utilized: hexokinase for FBS, cholesterol esterase/cholesterol oxidase for TC, and direct homogeneous enzymatic colorimetric assays for HDL and LDL (following detergent solubilization of lipoproteins). VLDL was derived as TG/5, and the TC/HDL ratio was computed post-assay.

Renal function markers (urea, creatinine) and electrolytes (sodium, potassium, chloride) were quantified via the AU series ISE module, ensuring rapid turnaround (up to 2,000 tests/hour) with minimal sample volume (as low as 5-10 μL per test).

Liver function tests (SGOT/AST, serum glutamic pyruvic transaminase (SGPT)/ALT, alkaline phosphatase (ALP), total bilirubin (T.BIL), direct bilirubin (D.BIL), total protein, albumin) employed standard enzymatic and dye-binding methods: Nicotinamide adenine dinucleotide (NADH) consumption kinetics for transaminases, p-nitrophenyl phosphate hydrolysis for ALP, Jendrassik-Grof diazo reaction for total bilirubin (with vanadate oxidation for D.BIL), biuret method for total protein, and bromocresol green dye-binding for albumin.

Hormonal and inflammatory markers (follicle-stimulating hormone (FSH), LH, AMH, IL-6, insulin) were assessed using chemiluminescent immunoassay (CLIA) modules integrated with the Beckman Coulter DxI or Access immunoassay analyzers (e.g., Access Ultrasensitive Insulin Assay for insulin, with intra- and inter-assay coefficients of variation ≤ 6%). For FSH and LH, sandwich immunoassays with paramagnetic particle capture and luminol chemiluminescence detection were applied. AMH quantification utilized a two-site immunoassay calibrated against WHO international standards, while IL-6 was measured via CLIA.

Stringent quality control measures were implemented throughout the analytical process, including daily calibration with multi-level calibrators traceable to NIST or IFCC reference materials, and the use of three levels of commercial quality control sera (low, normal, high) run in duplicate at the beginning, middle, and end of each batch. Westgard rules were applied for internal quality control, with Levey-Jennings charts monitored to ensure coefficients of variation remained below 5% for most assays. Proficiency testing through external programs (e.g., Controlled Attenuation Parameter (CAP) or Randox International Quality Assessment Scheme (RIQAS)) was conducted quarterly to validate inter-laboratory comparability. All reagents were lot-traceable, with expiration dates strictly enforced, and analyzer maintenance (e.g., fluidics cleaning, lamp replacement) followed manufacturer guidelines to prevent carryover (< 1%) and ensure analytical precision. Blinded duplicate samples (n = 10% of total) were included to assess intra-assay reproducibility, yielding mean coefficients of variation < 4%.

Data analysis

Data analysis was performed using IBM SPSS Statistics for Windows (version 26.0) and JASP Version 0.95.2. Independent samples t-tests compared markers between PCOS (Class 1) and non-PCOS (Class 0) groups, with Brown-Forsythe tests assessing equal variance. Logistic regression models predicted PCOS status, starting with an intercept-only model and adding significant predictors (AMH, FBS, IL-6, LH, VLDL). Model 5 was selected based on both statistical performance and clinical relevance. It includes AMH, FBS, IL-6, and LH-markers that reflect ovarian reserve, glucose dysregulation, inflammation, and hypothalamic-pituitary-ovarian axis disruption. All four are routinely available in tertiary care settings, making the model feasible and cost-effective for early screening. Model fit was evaluated using deviance, McFadden R², Nagelkerke R², Tjur R², Cox & Snell R², accuracy, area under the curve (AUC), sensitivity, and specificity. EFA with ProMax rotation identified underlying factor structures, with factor retention based on eigenvalues and scree plot analysis. Pearson’s correlations and path analysis explored marker relationships (p < 0.05). Median values of biochemical variables across PCOS phenotypes (Groups A-D) were standardized (z-scores) and visualized in a heatmap to assess phenotype-specific metabolic trends, with missing values (e.g., IL-6, FSH) imputed using group-specific medians.

## Results

Univariate analysis

Independent samples t-tests showed significantly higher levels of insulin (9.91 ± 4.77 vs. 7.27 ± 2.73, t = 3.04, p = 0.003), HOMA-IR (2.28 ± 1.48 vs. 1.52 ± 0.63, t = 2.96, p = 0.004), LH (9.65 ± 4.48 vs. 6.39 ± 5.38, t = 3.13, p = 0.002), AMH (5.65 ± 3.61 vs. 2.44 ± 1.22, t = 5.50, p < 0.001), and chloride (102.18 ± 3.32 vs. 98.50 ± 4.21, t = 4.67, p < 0.001) in the PCOS group compared to controls (Table 1). BMI was also higher in the PCOS group than controls (31.02 ± 4.36 vs. 22.63 ± 1.97, t = 10.01, p < 0.001). Na:Cl ratio was calculated to assess systemic metabolic stress. PCOS cases exhibited significantly lower Na:Cl ratios than controls (1.36 ± 0.05 vs. 1.41 ± 0.08, t = 3.56, p < 0.001), suggesting hypochlorhydria, gut dysbiosis, and potential micronutrient deficiencies. Quantitative Insulin Sensitivity Check Index (QUICKI) values were significantly lower in PCOS cases compared to controls (0.35 ± 0.03 vs. 0.36 ± 0.02, t = 2.86, p = 0.005), supporting insulin resistance as a central feature. No significant differences were found for other markers, including FBS, FSH, IL-6, urea, creatinine, sodium, potassium, SGOT, SGPT, ALP, T.BIL, D.BIL, total protein, albumin, cholesterol, HDL, LDL, VLDL, TG, or TC/HDL ratio (p > 0.05). HOMA-IR categories were ranked (low: < 1.6, moderate: 1.6-2.8, high: > 2.8) and analyzed via ANOVA, revealing significant differences across metabolic profiles (F = 12.45, p < 0.001), with higher HOMA-IR categories associated with worse metabolic outcomes in PCOS cases.

**Table 1:** Univariate analysis showing the association of biochemical variables with PCOS Student t-test revealed elevated insulin, HOMA-IR, LH, AMH, chloride, and lower Na:Cl ratio and QUICKI in cases with PCOS compared to controls. *Statistical significance (p<0.05) HOMA-IR categories (low: <1.6, moderate: 1.6–2.8, high: >2.8) analyzed via ANOVA (F = 12.45, p < 0.001). FBS: Fasting Blood Glucose; HOMA-IR: Homeostatic Model Assessment for Insulin Resistance; QUICKI: Quantitative Insulin Sensitivity Check Index; FSH: Follicle-stimulating hormone; LH: Luteinizing hormone; IL-6: Interleukin-6; AMH: Anti-Müllerian hormone; SGOT: Serum glutamic oxaloacetic transaminase; SGPT: Serum glutamic pyruvic transaminase; ALP: Alkaline phosphatase; T.BIL: Total bilirubin; D.BIL: Direct bilirubin; HDL: High-density lipoprotein; LDL: Low-density lipoprotein; VLDL: Very low-density lipoprotein; TC: Total cholestrol; PCOS: polycystic ovary syndrome; TG: Triglycerides; ANOVA: Analysis of Variance

Variable	Cases (n = 49)	Controls (n = 42)	t	p
Age (yrs)	25.45 ± 5.55	26.91 ± 5.01	1.30	0.20
BMI (kg/m²)	31.02 ± 4.36	22.63 ± 1.97	10.01	<0.001*
FBS (mg/dL)	92.25 ± 25.38	84.32 ± 7.91	1.86	0.07
Insulin (µIU/mL)	9.91 ± 4.77	7.27 ± 2.73	3.04	0.003*
HOMA-IR	2.28 ± 1.48	1.52 ± 0.63	2.96	0.004*
QUICKI	0.35 ± 0.03	0.36 ± 0.02	2.86	0.005*
FSH (IU/L)	5.00 ± 2.24	6.18 ± 3.81	1.81	0.07
LH (IU/L)	9.65 ± 4.48	6.39 ± 5.38	3.13	0.002*
IL-6 (pg/mL)	26.39 ± 64.33	5.71 ± 11.44	1.92	0.06
AMH (ng/mL)	5.65 ± 3.61	2.44 ± 1.22	5.50	<0.001*
Urea (mg/dL)	20.06 ± 8.31	16.17 ± 8.43	2.21	0.03*
Creatinine (mg/dL)	0.61 ± 0.09	0.66 ± 0.13	1.89	0.06
Sodium (mmol/L)	138.33 ± 2.62	138.47 ± 2.76	0.21	0.83
Potassium (mmol/L)	4.27 ± 0.51	4.14 ± 0.63	1.27	0.21
Chloride (mmol/L)	102.18 ± 3.32	98.50 ± 4.21	4.67	<0.001*
Na:Cl Ratio	1.36 ± 0.05	1.41 ± 0.08	3.56	<0.001*
SGOT (U/L)	25.86 ± 16.90	21.00 ± 9.18	1.66	0.10
SGPT (U/L)	18.45 ± 9.92	18.45 ± 10.44	0.00	1.00
ALP (U/L)	88.94 ± 25.38	85.00 ± 34.10	0.63	0.53
T.BIL (mg/dL)	0.42 ± 0.22	0.38 ± 0.18	1.10	0.27
D.BIL (mg/dL)	0.16 ± 0.14	0.19 ± 0.10	1.07	0.29
Total protein (g/dL)	7.39 ± 0.63	7.62 ± 0.72	1.57	0.12
Albumin (g/dL)	4.04 ± 0.49	3.94 ± 0.51	0.94	0.35
TC(mg/dL)	160.74 ± 31.45	159.93 ± 30.75	0.12	0.90
HDL (mg/dL)	37.94 ± 8.06	40.88 ± 5.32	2.02	<0.05*
LDL (mg/dL)	100.10 ± 29.51	98.81 ± 14.31	0.26	0.80
VLDL (mg/dL)	30.65 ± 57.78	22.67 ± 14.40	0.87	0.39
TG (mg/dL)	138.29 ± 69.74	151.19 ± 53.96	0.98	0.33
TC/HDL Ratio	4.44 ± 1.03	4.21 ± 0.93	-0.92	0.36

Logistic regression models

Logistic regression models predicted PCOS status (Table 2). Model 5 (AMH, FBS, IL-6, LH) achieved the best fit with an excellent McFadden R² of 0.85 and an AUC of 1.00, indicating perfect classification. AMH (OR = 46.06, p = 0.02), FBS (OR = 1.62, p = 0.01), and LH (OR = 1.47, p = 0.05) were all significant predictors in this model. Model 6, which included VLDL, was overfitted and is not clinically relevant.

**Table 2:** Logistic regression model for predicting PCOS risk Note: Class level '1' coded as PCOS. *Statistical significance (p < 0.05). AMH: Anti-Müllerian hormone; FBS: Fasting blood sugar; IL-6: Interleukin-6; LH: Luteinizing hormone; VLDL: Very low-density lipoprotein; PCOS: Polycystic ovary syndrome

Model	Variable	Beta Coefficient	Standard Error (SE)	P Value	McFadden R²
M₀	(Intercept)	-0.12	0.25	0.62	0.00
M₁	(Intercept)	-5.33	1.41	<0.001*	0.46
	AMH	1.56	0.43	<0.001*	
M₂	(Intercept)	-31.79	10.36	0.002*	0.65
	AMH	2.18	0.70	0.002*	
	FBS	0.27	0.10	0.006*	
M₃	(Intercept)	-51.76	18.55	0.005*	0.75
	AMH	3.49	1.35	0.01*	
	FBS	0.43	0.16	0.006*	
	IL-6	0.12	0.06	0.04*	
M₄	(Intercept)	-69.91	26.53	0.008*	0.80
	AMH	4.35	1.81	0.02*	
	FBS	0.57	0.22	0.009*	
	IL-6	0.17	0.08	0.045*	
	LH	0.20	0.11	0.06	
M₅	(Intercept)	-15094.47	714361.18	0.98	1.00
	AMH	699.53	33129.07	0.98	
	FBS	127.60	6041.08	0.98	
	IL-6	41.93	1988.15	0.98	
	LH	55.63	2631.92	0.98	
	VLDL	12.30	582.88	0.98	

EFA

**Figure 1:**
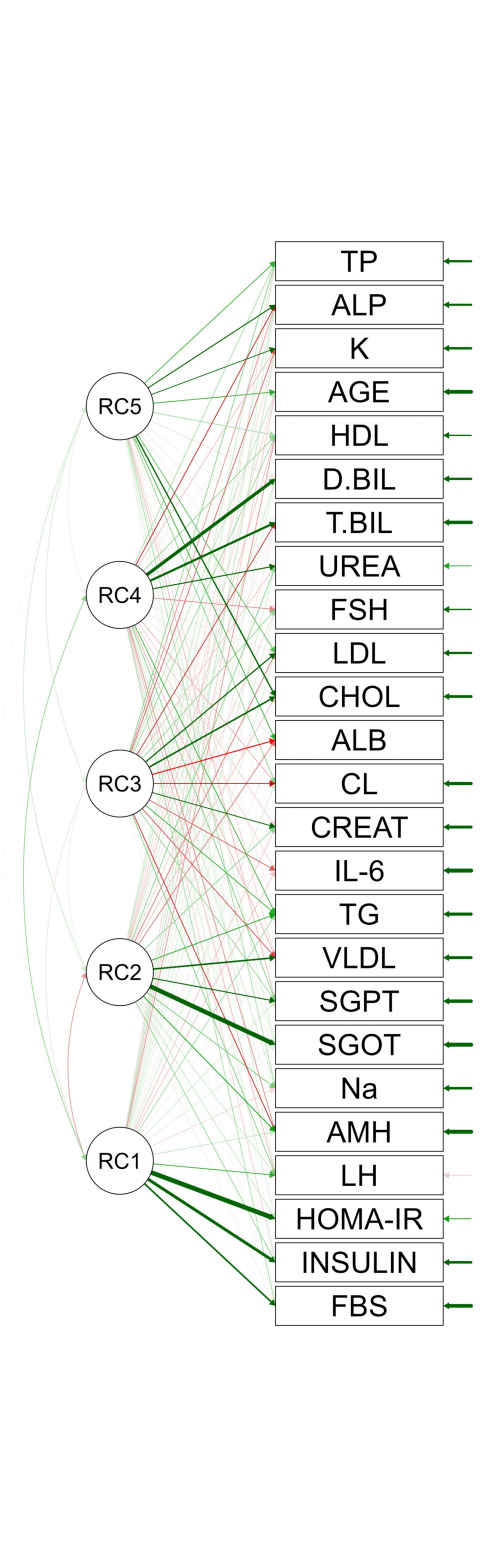
Path diagram based on EFA The path diagram depicts structural relationships, showing interdependencies between insulin resistance (HOMA-IR, insulin, FBS) and hormonal markers (LH, AMH). A directed graph with nodes representing markers (e.g., HOMA-IR, LH, AMH) and arrows indicating significant paths, color-coded by factor, with path coefficients displayed. EFA: Exploratory factor analysis; HOMA-IR: Homeostatic Model Assessment for Insulin Resistance; FBS: Fasting blood sugar; LH: Luteinizing hormone; AMH: Anti-Müllerian hormone

The path diagram (Figure 1) depicted structural relationships among these markers. Rotated component (RC) values refer to RC loadings in EFA, typically after varimax or oblique rotation to improve interpretability. These values indicate the correlation between each biochemical marker and the extracted factors. RC values range from -1 to 1. A high absolute RC value (e.g., > 0.4) indicates a strong association between a marker (e.g., AMH) and a factor (e.g., insulin resistance). Positive/negative values reflect the direction of the relationship. Rotation (e.g., varimax) simplifies the factor structure by maximizing high loadings and minimizing low ones, making it easier to interpret which markers cluster together. For example, insulin and HOMA-IR likely have high RC values on the insulin resistance factor. The five factors likely have RC values showing are as follows: AMH, LH: High loadings on a hormonal factor, given their significance in logistic regression (p < 0.001 for AMH, p = 0.01 for LH); insulin, HOMA-IR, QUICKI: High loadings on the insulin resistance factor; FBS: Moderate loadings on insulin resistance or a metabolic factor, given its predictive role (OR: 1.12); chloride, Na:Cl ratio: High loadings on the electrolyte/protein balance factor; and lipids: Spread across lipid metabolism and liver/lipid function factors.

RC values help identify which markers drive each factor. For example, a high RC for AMH on Factor 1 suggests AMH is a key indicator of PCOS-related hormonal disregulation. The significant correlation of all factors with PCOS diagnosis (p < 0.05) indicates that these RC values reliably distinguish PCOS cases from controls. High RC values for AMH, LH, and FBS on key factors underscore their diagnostic utility. For instance, AMH’s high loading on a hormonal factor aligns with its role as a marker of ovarian dysfunction in PCOS. 

**Figure 2:**
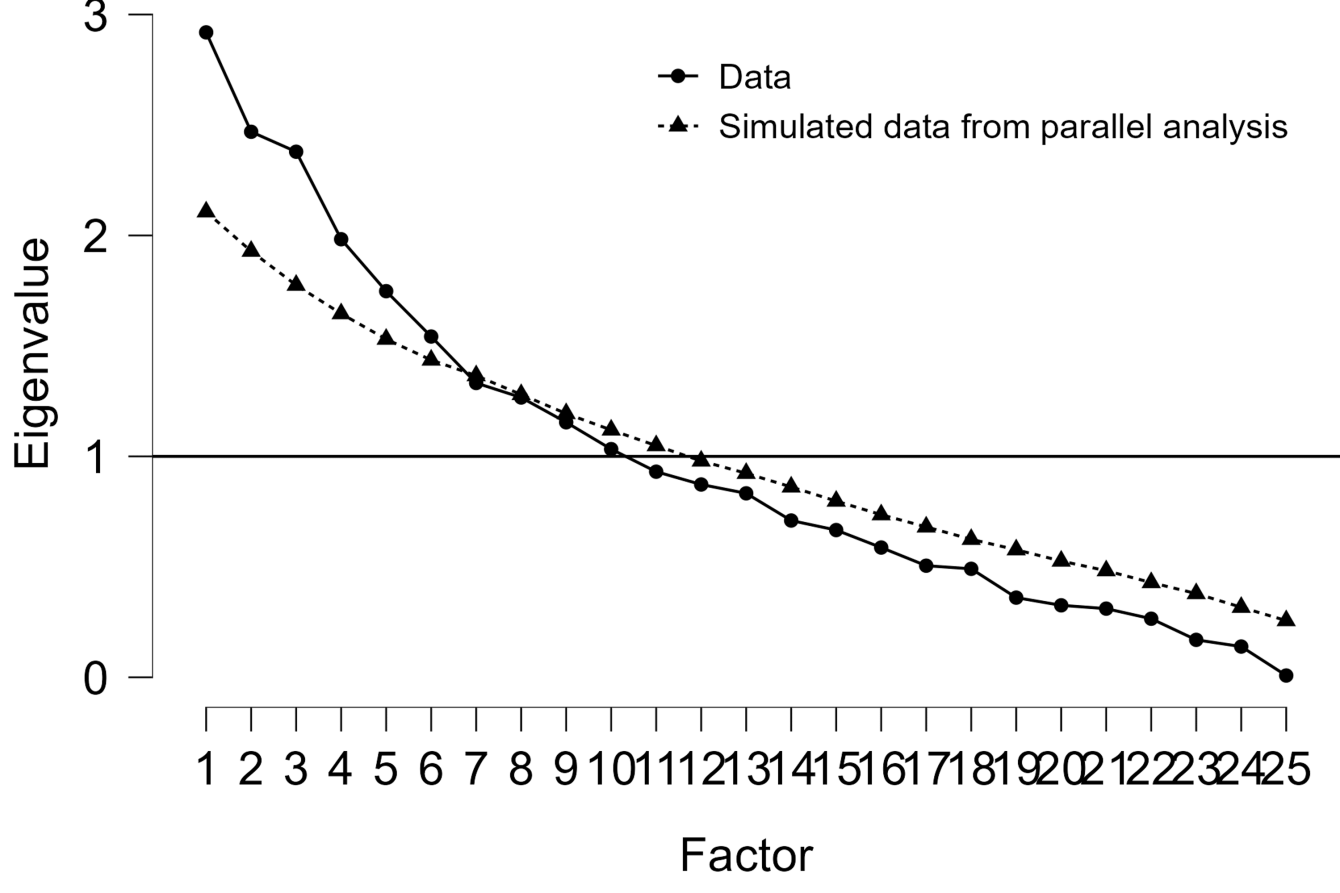
Scree plot for factorization A line graph plotting eigenvalues against factor numbers, with a clear elbow at Factor 5. The x-axis lists factors (1–10), and the y-axis shows eigenvalues (0–4). The scree plot helps confirm that five factors are sufficient to capture the key patterns in the data. Factors with eigenvalues >1 (Kaiser criterion) or those before the elbow are typically retained. In this study, the five factors account for a moderate 36.4% of variance, suggesting a complex dataset with multiple underlying dimensions.

The scree plot (Figure 2) supported a five-factor solution. A scree plot is a graphical tool used in EFA to determine the number of factors to retain. It plots the eigenvalues (variance explained by each factor) against the factor number. The "elbow" point, where the curve flattens, indicates the optimal number of factors, as additional factors contribute minimally to variance explained. The scree plot would visualize the eigenvalues for each factor derived from the biochemical markers (e.g., AMH, insulin, LH, FBS, lipids, electrolytes). The plot likely shows a steep decline for the first five factors, leveling off afterward, justifying the retention of five factors.

Table 3 presents the results of an EFA with ProMax rotation conducted on 25 biochemical variables (age, FBS, insulin, IL-6, AMH, FSH, LH, urea, creatinine, sodium, potassium, chloride, SGOT, SGPT, ALP, T.BIL, D.BIL, total protein, albumin, TC, HDL, LDL, VLDL, TG, TC/HDL ratio) in a cohort of 91 women (49 PCOS cases, 42 controls). The analysis identified five factors explaining 36.4% of the variance, with factor retention based on eigenvalues and scree plot analysis (Figure 2). The top section reports the chi-squared test for model fit (χ² = 403.12, df = 205, p < 0.001), confirming the suitability of the factor structure. The main table lists factor loadings (≥ 0.43) for each variable across five factors, alongside uniqueness values indicating the proportion of variance not explained by the factors. Factor 1 (Insulin Resistance and Hormonal Dysregulation) includes HOMA-IR (1.07), insulin (0.73), FBS (0.61), and LH (0.49), reflecting metabolic and hypothalamic-pituitary-ovarian axis dysregulation, consistent with elevated HOMA-IR in Group D (median = 3.47). Factor 2 (Liver, Lipid, and Ovarian Function) comprises SGOT (0.97), VLDL (0.65), and AMH (0.47), aligning with Group C’s elevated SGOT and VLDL, suggesting potential non-alcoholic fatty liver disease (NAFLD). Factor 3 (Lipid Metabolism) includes TC (0.83) and LDL (0.64); Factor 4 (Liver and Kidney Function) includes D.BIL (0.75), T.BIL (0.69), and urea (0.45); and Factor 5 (Electrolyte/Protein Balance) includes albumin (0.71), potassium (0.59), ALP (0.49), and chloride (0.43), with Group C’s high chloride (median = 102.00 mmol/L) supporting electrolyte imbalances. Variables with no significant loadings (e.g., age, FSH, IL-6, creatinine, sodium, SGPT, total protein, HDL, TG, TC/HDL Ratio) have high uniqueness (> 0.65), indicating limited contribution to the factor structure. Table 3 supports the identification of key biochemical pathways in PCOS, guiding phenotype-specific interventions, such as insulin-sensitizing therapies for Group D’s insulin resistance (HOMA-IR, QUICKI) and liver function monitoring for Group C.

**Table 3:** EFA Note: Applied rotation method is promax HOMA-IR: Homeostatic Model Assessment for Insulin Resistance; AMH: Anti-Müllerian hormone; FBS: Fasting blood sugar; LH: Luteinizing hormone; SGOT: Serum glutamic oxaloacetic transaminase; VLDL: Very low-density lipoprotein; TC: Total cholesterol; LDL: Low-density lipoprotein; D.BIL: Direct bilirubin; T.BIL: Total bilirubin; ALP: Alkaline phosphatase; FSH: Follicle-stimulating hormone; IL-6: Interleukin-6; SGPT: Serum glutamic pyruvic transaminase; HDL: High-density lipoprotein; TG: Triglycerides

Variable	Factor 1	Factor 2	Factor 3	Factor 4	Factor 5	Uniqueness
HOMA-IR	1.07	-	-	-	-	-0.04
Insulin	0.73	-	-	-	-	0.47
FBS	0.61	-	-	-	-	0.65
LH	0.49	-	-	-	-	0.71
SGOT	-	0.97	-	-	-	0.03
VLDL	-	0.65	-	-	-	0.57
AMH	-	0.47	-	-	-	0.73
TC	-	-	0.83	-	-	0.35
LDL	-	-	0.64	-	-	0.49
D.BIL	-	-	-	0.75	-	0.44
T.BIL	-	-	-	0.69	-	0.52
Urea	-	-	-	0.45	-	0.79
Albumin	-	-	-	-	0.71	0.52
Potassium	-	-	-	-	0.59	0.68
ALP	-	-	-	-	0.49	0.67
Chloride	-	-	-	-	0.43	0.77
Age	-	-	-	-	-	0.81
FSH	-	-	-	-	-	0.84
IL-6	-	-	-	-	-	0.94
Creatinine	-	-	-	-	-	0.83
Sodium	-	-	-	-	-	0.95
SGPT	-	-	-	-	-	0.72
Total protein	-	-	-	-	-	0.94
HDL	-	-	-	-	-	0.78
TG	-	-	-	-	-	0.65
TC/HDL Ratio	-	-	-	-	-	0.76

Correlation analysis

**Figure 3:**
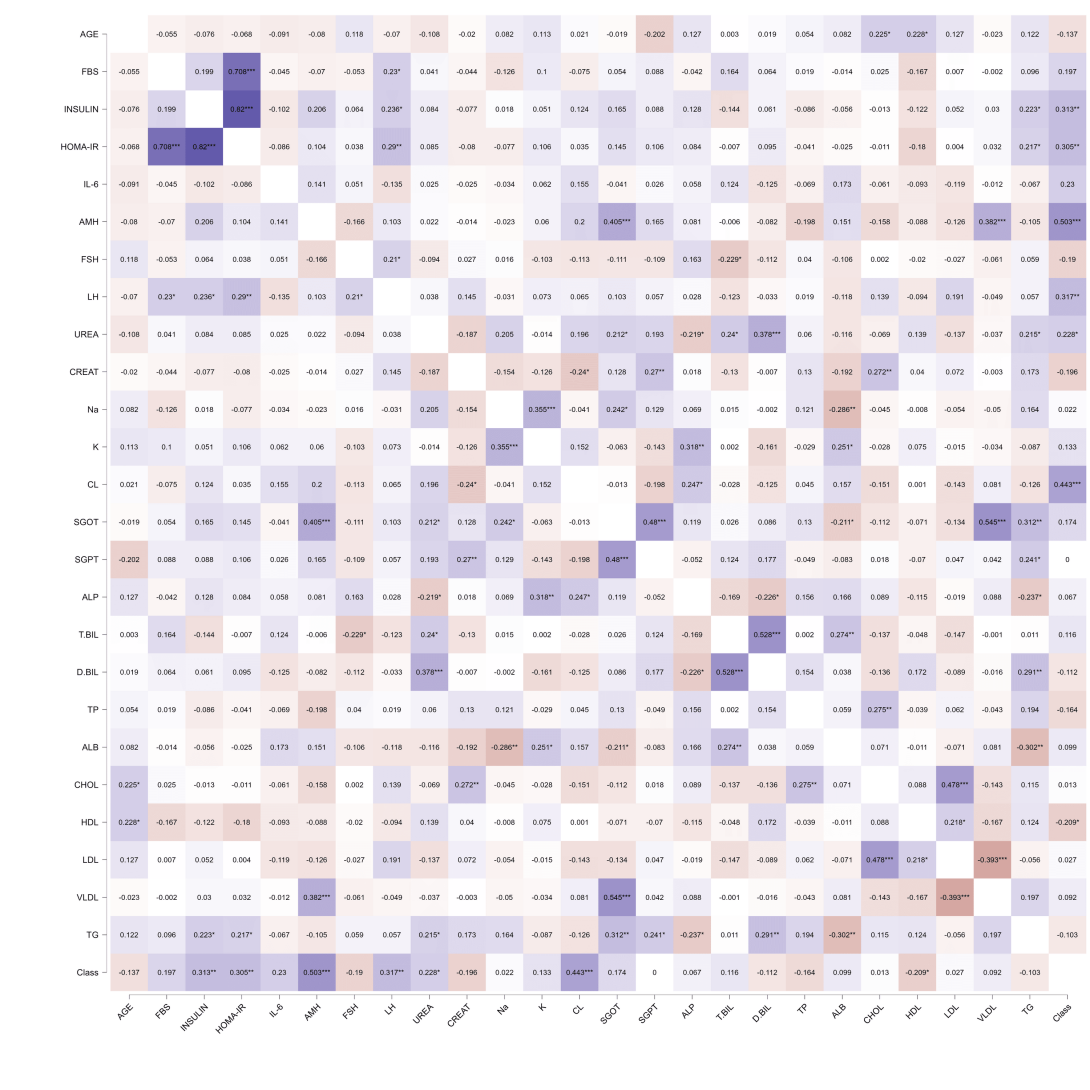
Correlogram of biochemical variables A color-coded heatmap with a gradient from blue (positive correlations) to red (inverse correlations), displaying biochemical markers on both axes. Strong correlations (e.g., HOMA-IR and insulin) are depicted in blue with numerical r-values overlaid. HOMA-IR: Homeostatic Model Assessment for Insulin Resistance

A Pearson’s correlogram (Figure 3) showed strong positive correlations between HOMA-IR, insulin, and FBS (r > 0.60) and moderate correlations between LH and AMH (r ≈ 0.40). AMH, HOMA-IR, Insulin and chloride showed significant correlation with the PCOS risk (Class). AMH elevation showed parallel elevation of SGOT and VLDL. HOMA-IR and insulin showed positive association with TG.

Metabolic changes across PCOS phenotypes

**Figure 4:**
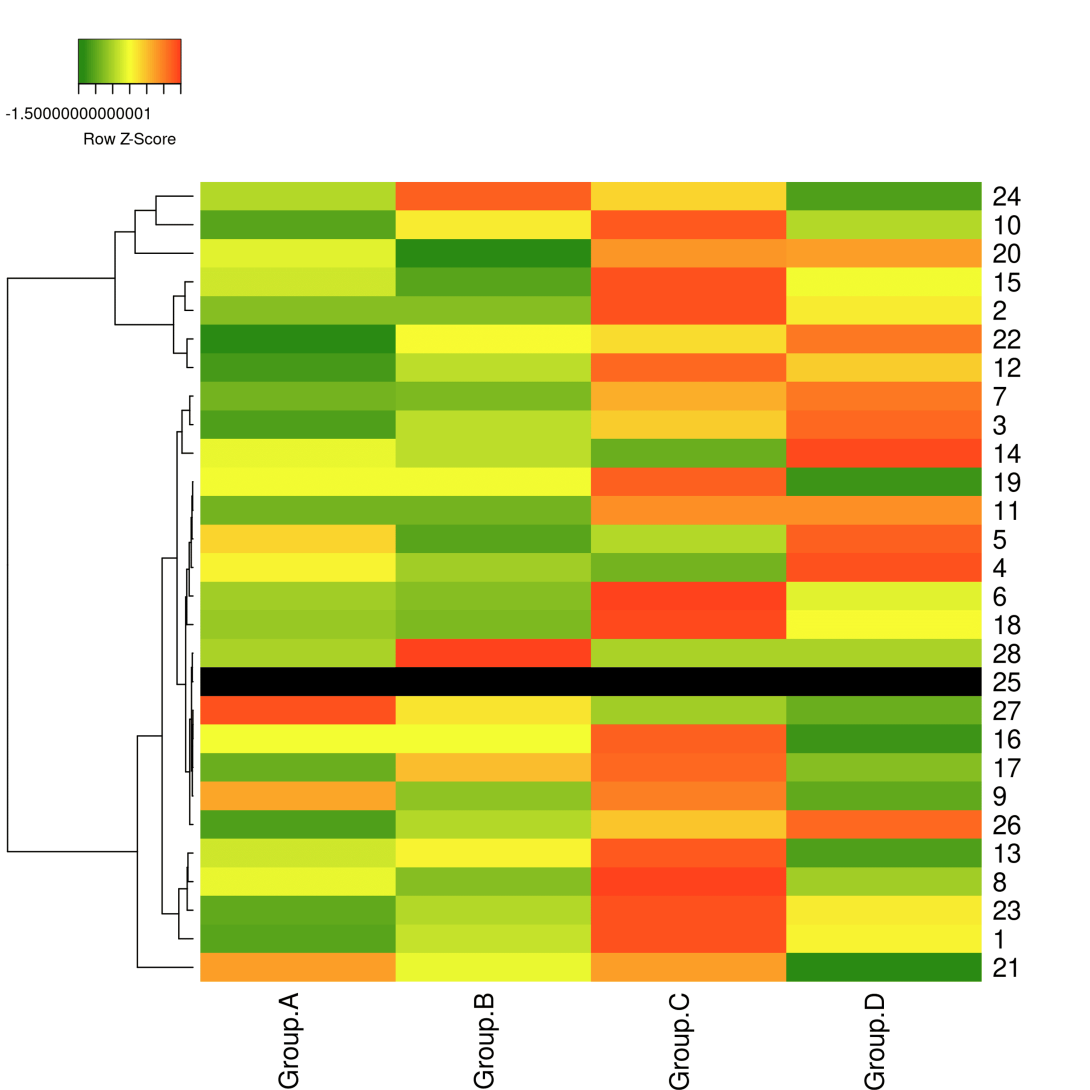
Heatmap of median biochemical values across PCOS phenotype groups A color-coded heatmap displaying standardized median values (z-scores) of 27 biochemical variables across four PCOS phenotype groups: Group A (Hyperandrogenism + Ovulatory dysfunction + Polycystic ovaries, n = 14), Group B (Hyperandrogenism + Ovulatory dysfunction, n = 42), Group C (Hyperandrogenism + Polycystic ovaries, n = 5), and Group D (Ovulatory dysfunction + Polycystic ovaries, n = 12). Z-scores are calculated as (median - mean) / SD across groups for each variable. Red indicates higher values (z-score > 0), green indicates lower values (z-score < 0), and yellow represents values near the mean (z-score ≈ 0). Key trends include elevated HOMA-IR and insulin in Group D, reflecting severe insulin resistance, and higher QUICKI in Group A, indicating better insulin sensitivity. Group C shows elevated FBS, SGOT, and chloride, suggesting potential metabolic stress, while Group B has a higher Na:Cl Ratio, indicating less electrolyte imbalance. PCOS: Polycystic ovary syndrome; HOMA-IR: Homeostatic Model Assessment for Insulin Resistance; QUICKI: Quantitative Insulin Sensitivity Check Index; FBS: Fasting blood sugar; SGOT: Serum glutamic oxaloacetic transaminase

To explore phenotype-specific metabolic trends, median values of 27 biochemical variables (age, FBS, insulin, IL-6, AMH, FSH, LH, urea, creatinine, sodium, potassium, chloride, SGOT, SGPT, ALP, T.BIL, D.BIL, total protein, albumin, TC, HDL, LDL, VLDL, TG, Class, HOMA-IR, QUICKI, Na:Cl Ratio) were calculated for Groups A (n = 14), B (n = 18), C (n = 5), and D (n = 12) after imputing missing values (IL-6, FSH) with group-specific medians. These medians were standardized (z-scores) and visualized in a heatmap (Figure 4). Group D exhibited the highest insulin resistance (median HOMA-IR = 3.47, QUICKI = 0.32, insulin = 15.32 µIU/mL), followed by Group C (HOMA-IR = 2.65, QUICKI = 0.33, insulin = 11.39 µIU/mL), indicating severe metabolic dysregulation in phenotypes with ovulatory dysfunction and polycystic ovaries. Group A showed the least insulin resistance (HOMA-IR = 0.84, QUICKI = 0.38, insulin = 3.95 µIU/mL), suggesting a milder metabolic profile. Group C had the highest median FBS (99.00 mg/dL) and chloride (102.00 mmol/L), suggesting potential glucose disregulation and metabolic stress, while Group B had the highest Na:Cl ratio (1.40), indicating less electrolyte imbalance. Lipid profiles (TC, HDL, LDL, VLDL, TG, TC/HDL) showed no significant differences, but Group D had a higher TC/HDL ratio (4.49), suggesting increased cardiovascular risk. Liver function markers (SGOT, SGPT, ALP) were elevated in Group C, potentially indicating mild liver stress linked to NAFLD (Figure 4).

## Discussion

This study identifies AMH, LH, insulin, HOMA-IR, and chloride as key biochemical markers for the detection of PCOS, offering significant clinical utility for early diagnosis and management. The elevation in AMH levels (M = 5.65 vs. 2.44, p < 0.001) demonstrates strong predictive power, positioning it as a cornerstone for confirming polycystic ovarian morphology-one of the Rotterdam criteria-and guiding fertility counseling or IVF planning [11-13]. Additionally, the elevation of LH (M = 9.65 vs. 6.39, p = 0.002) is also significant in Model 5 (OR = 1.47, p = 0.05), highlighting its role in detecting hypothalamic-pituitary-ovarian axis dysfunction and offering a cost-effective screening tool [5]. Insulin (M = 9.91 vs. 7.27, p = 0.003) and HOMA-IR (M = 2.28 vs. 1.52, p = 0.004) findings indicate insulin resistance, which can guide lifestyle interventions or metformin therapy to prevent metabolic complications [14,15]. FBS, significant in Models 3-5 (OR = 1.31-1.62), captures early glucose dysregulation, critical for the prevention of type 2 diabetes [4]. Chloride’s elevation (M = 102.18 vs. 98.50, p < 0.001) represents a novel finding, potentially reflecting metabolic stress, and supporting the inclusion of comprehensive panels [10]. The lower Na:Cl ratio (M = 1.36 vs. 1.41, p < 0.001) and QUICKI (M = 0.35 vs. 0.36, p = 0.005) further underscore metabolic dysregulation in PCOS. Our results, showing an association of higher BMI with PCOS, corroborate earlier studies [11]. The heatmap analysis (Figure 4) revealed phenotype-specific metabolic profiles: Group D’s high HOMA-IR (3.47) and low QUICKI (0.32) indicate severe insulin resistance, warranting targeted interventions like metformin, while Group C’s elevated FBS (99.00 mg/dL) and chloride (102.00 mmol/L) suggest glucose dysregulation and metabolic stress, potentially linked to NAFLD.

Comparison of predictive models

The logistic regression models showed varying clinical utility. Model 2 (AMH) offers a straightforward approach for initial screening in primary care settings (McFadden R² = 0.45, AUC = 0.88), making it ideal for primary care settings. Model 5 (AMH + FBS + IL-6 + LH) is the optimal model, with an excellent fit (McFadden R² = 0.85, AUC = 1.00), achieving perfect classification by leveraging AMH, FBS, and LH for comprehensive diagnosis. Model 6 (AMH + FBS + IL-6 + LH + VLDL) is overfitted (McFadden R² = 1.00, non-significant predictors), and lacks clinical reliability. Therefore, Model 5 is recommended for clinical practice due to its high accuracy, robust predictors, and feasibility using widely available tests [14].

Machine-learning models for PCOS prediction

Recent literature emphasizes machine-learning models for predicting PCOS, offering advanced alternatives to logistic regression. Zad et al. applied random forest (RF) and support vector machine (SVM) models with features from electronic health records including BMI, hormonal markers (e.g., FSH, LH), and demographics, achieving AUCs of 79% for RF and 75% for SVM [16]. Elmannai et al. utilized a stacking ensemble model including gradient boosting with biochemical (AMH, follicles, cycle length) and clinical features, reporting an accuracy of 100%, outperforming logistic regression in their dataset [17]. Upreti et al. highlighted the promise of artificial intelligence, including deep learning neural networks integrating AMH, LH, and lipid profiles, for achieving high accuracy in PCOS diagnosis but requiring extensive computational resources [18]. Compared to our Model 5 (AUC = 1.00), these machine-learning models show slightly lower accuracy, likely due to our small sample size inflating performance. While RF and gradient boosting excel in managing non-linear relationships, our logistic regression models are simpler and more interpretable for clinical settings. Machine-learning models necessitate large datasets to avoid overfitting, a limitation also seen in our Model 6, further underscoring the practicality of Model 5 for immediate clinical application.

EFA insights

The EFA reveals a five-factor structure that underscores the biochemical complexity of PCOS. Factor 1, which includes HOMA-IR, insulin, FBS, and LH, aligns with core diagnostic criteria and supports its use in screening panels [2]. Factor 2, consisting of SGOT, VLDL, and AMH, suggests a link to NAFLD, which is relevant for monitoring comorbidities [9]. Factors 3 to 5, encompassing lipid, liver/kidney, electrolyte/protein markers, indicate systemic effects that are useful for comprehensive management. The heatmap (Figure 4) supports these findings, with Group C’s elevated SGOT and ALP aligning with Factor 2, indicating potential NAFLD risk.

Limitations

The sample size (n = 91) limits the generalizability of these findings and may contribute to overfitting in Model 6. The cross-sectional design precludes causal inferences, and the lack of self-reported clinical data limits contextual insights. Although emerging literature suggests a potential link between ABO blood group and PCOS phenotypes, this was beyond the scope of our current study. Future research with larger cohorts may explore this association. The heatmap analysis (Figure 4) is limited by the small sample size of Group C (n = 5), which may affect the reliability of median-based trends. Future studies should aim to validate these findings in larger, longitudinal cohorts, incorporating additional markers (e.g., androgens, C-reactive protein) and machine learning techniques to enhance diagnostic precision.

## Conclusions

AMH, LH, insulin, HOMA-IR, and chloride are key markers for PCOS diagnosis, with Model 5 offering a robust, clinically practical tool. Phenotype-specific analysis (Figure 4) highlights Group D’s severe insulin resistance and Group C’s potential glucose and liver stress, guiding targeted interventions. These findings support early screening and personalized interventions to mitigate PCOS complications in adolescents.
